# Quantitative Analysis of Porous Silicon Nanoparticles Functionalization by ^1^H NMR

**DOI:** 10.1021/acsbiomaterials.1c00440

**Published:** 2021-07-22

**Authors:** Ruoyu Cheng, Shiqi Wang, Karina Moslova, Ermei Mäkilä, Jarno Salonen, Jiachen Li, Jouni Hirvonen, Bing Xia, Hélder A. Santos

**Affiliations:** ‡ Drug Research Program, Division of Pharmaceutical Chemistry and Technology, Faculty of Pharmacy, University of Helsinki, Helsinki FI-00014, Finland; § Helsinki Insititute of Life Science, HiLIFE, University of Helsinki, Helsinki FI-00014, Finland; ⊥ Department of Chemistry, Faculty of Science, University of Helsinki, Helsinki FI-00014, Finland; || College of Science Key Laboratory of Forest Genetics & Biotechnology (Ministry of Education of China), Nanjing Forestry University, Nanjing 210037, P. R. China; # Laboratory of Industrial Physics, Department of Physics and Astronomy, University of Turku, Turku FI-20014, Finland

**Keywords:** porous silicon, nanoparticles, quantitative NMR, surface chemistry

## Abstract

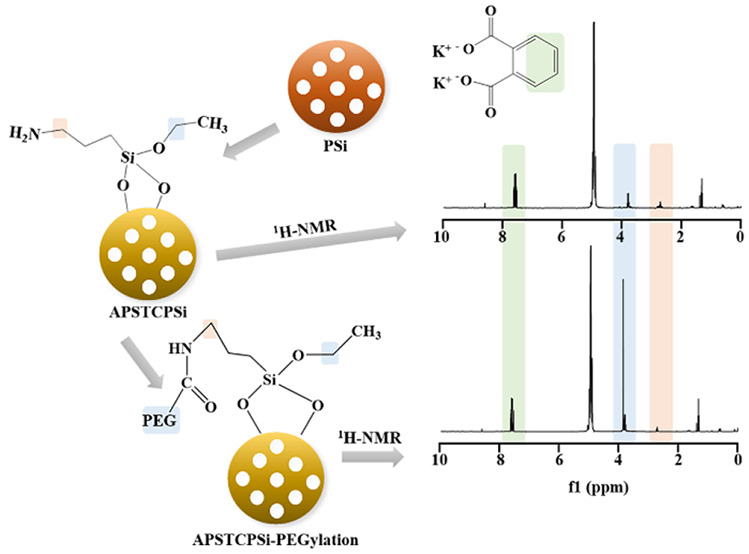

Porous silicon (PSi) nanoparticles have been applied in various fields, such as catalysis, imaging, and biomedical applications, because of their large specific surface area, easily modifiable surface chemistry, biocompatibility, and biodegradability. For biomedical applications, it is important to precisely control the surface modification of PSi-based materials and quantify the functionalization density, which determines the nanoparticle’s behavior in the biological system. Therefore, we propose here an optimized solution to quantify the functionalization groups on PSi, based on the nuclear magnetic resonance (NMR) method by combining the hydrolysis with standard ^1^H NMR experiments. We optimized the hydrolysis conditions to degrade the PSi, providing mobility to the molecules for NMR detection. The NMR parameters were also optimized by relaxation delay and the number of scans to provide reliable NMR spectra. With an internal standard, we quantitatively analyzed the surficial amine groups and their sequential modification of polyethylene glycol. Our investigation provides a reliable, fast, and straightforward method in quantitative analysis of the surficial modification characterization of PSi requiring a small amount of sample.

Porous silicon (PSi) was accidentally discovered by Uhlir while searching for a technique to shape the surface of silicon.^1^ During the following decades, scientists have proved the quantum confinement effects of PSi with efficient visible photoluminescence, nonlinear optical and electrical properties.^1^ In 1995, Canham confirmed that PSi is biodegradable. The degradation product is the nontoxic orthosilicic acid [Si (OH)_4_], a natural compound found in the human body.^2,3^ Consequently, and thereafter, PSi has been widely explored for biomedical applications, such as bioimaging, drug carriers for targeting delivery, and vaccine adjuvants for cancer immunotherapy.^4−12^

PSi nanoparticles for biomedical applications are usually functionalized with targeting ligands, stimuli-responsive moieties, and polyethylene glycol (PEG), to improve the biodistribution at the target site, control the payload release, and increase the stability, respectively.^13,14^ After the surface modification, it is essential to quantify the modification efficiency on the PSi’s surface, because the surface modification density will influence the PSi behavior.^15^ However, it is technically challenging to quantify the exact number of functional groups on the PSi’s surface or the density of conjugated molecules, requiring the detection of small quantities of organic molecules on solid inorganic nanoparticles.

Currently, the common quantitative analysis methods include elemental analysis, thermogravimetric analysis (TGA), and colorimetric reactions. For example, Ferreira et al. analyzed the amount of atrial natriuretic peptide conjugated at the surface of PSi by elemental analysis.^16^ Because the nitrogen and sulfur element is only present in the peptide rather than in the PSi matrix, the conjugated peptide quantity can be calculated accordingly. Kunc et al. quantitatively analyzed the surface hydroxyl functional groups on silica (SiO_2_) nanoparticles by TGA, but this method is suitable for those with the large mass fraction of functional groups (e.g., polymers).^17^ Similarly, Sun et al. quantitated the polymethacrylate on the surface of solid silicon nitride nanoparticles by TGA.^18^

Despite being successfully used in literature, the above analysis methods exhibit different limitations. For example, the test samples have to be dry for the TGA analysis. The initial weight loss is subjected to sample fabrication and drying methods.^17^ In addition, the thermal condensation of hydroxyl on the surface of PSi can interfere with the decomposition peaks of organic compositions, complicating the quantitative analysis, as previously reported in silica nanoparticles.^19,20^ As for the elemental analysis, it is only applicable to functionalization with specific additional elements, because the results reflect the elemental compositions in the sample, instead of specific functional moieties. For colorimetric reactions, there must be chromogenic, fluorescent, or luminescent compounds in the analysis samples. Although colorimetric assays are relatively sensitive, the innate color, fluorescence, or luminescence from PSi nanoparticles should be taken into consideration in such assays, because it can influence the quantitative results.^21^ In these cases, it will be difficult to calculate the functionalization degree and how it will affect the particle behavior in the biological system. Thus, it is desirable to develop a highly sensitive complementary analysis method without complicated processes for the quantitative analysis of the surface modification of PSi.

Quantitative NMR (qNMR) has been widely used for the determination of concentration and purity of small molecules because of its high sensitivity, high reproducibility, and robustness.^22^ Different from the solid-state (SS) NMR that usually needs a large amount of dried powder sample and results in broad peaks, the solution qNMR method exhibits excellent spectral resolution.^23^ Recently, there have been a few reports about using the solution qNMR for quantification of SiO_2_ nanoparticles after fully dissolving the particles in alkaline solution.^24,25^ After the dissolution of the silica matrix, the functionalized molecules previously attached to the particle’s surface have increased mobility, making them easily detectable. This method is applicable to both solid and porous SiO_2_ nanoparticles with different surface functionalities, and the results correlate well with conventional quantification methods (TGA and colorimetric assays).^24,26^ Moreover, qNMR can analyze multiple organic molecules at the same time, providing the critical peaks are not overlapping in the spectrum.^24^

Inspired by these previous studies, we developed a quantitative analysis method for the PSi’s surface chemistry by solution ^1^H NMR (Scheme 1). We chose (3-aminopropyl) triethoxysilane (APS) functionalized thermally carbonized PSi nanoparticles (APSTCPSi), which have been widely used in biomedical applications.^27,28^ The hydrodynamic diameter and zeta-potential of the APSTCPSi nanoparticles were evaluated by a Zetasizer Nano ZS (Figure 1A, B). The hydrodynamic size was 224 ± 6 nm, and the polydispersity index (PDI) was 0.11 ± 0.01 indicating the homogeneous distribution of APSTCPSi nanoparticles. The surface zeta-potential was +33 ± 2 mV, indicating the successful surface modification of the APSTCPSi nanoparticles PSi with amine groups. Moreover, the morphology of the APSTCPSi nanoparticles was analyzed by a transmission electron microscope (TEM, Figure 1C). The porous structures showed the successful synthesis of APSTCPSi nanoparticles, verified by the nitrogen sorption results (Figure S1), including a specific surface area of 288 ± 1 m^2^/g, pore volume of 0.76 ± 0.05 cm^3^/g, and an average pore diameter of 10.5 ± 0.6 nm.

**Scheme 1:**
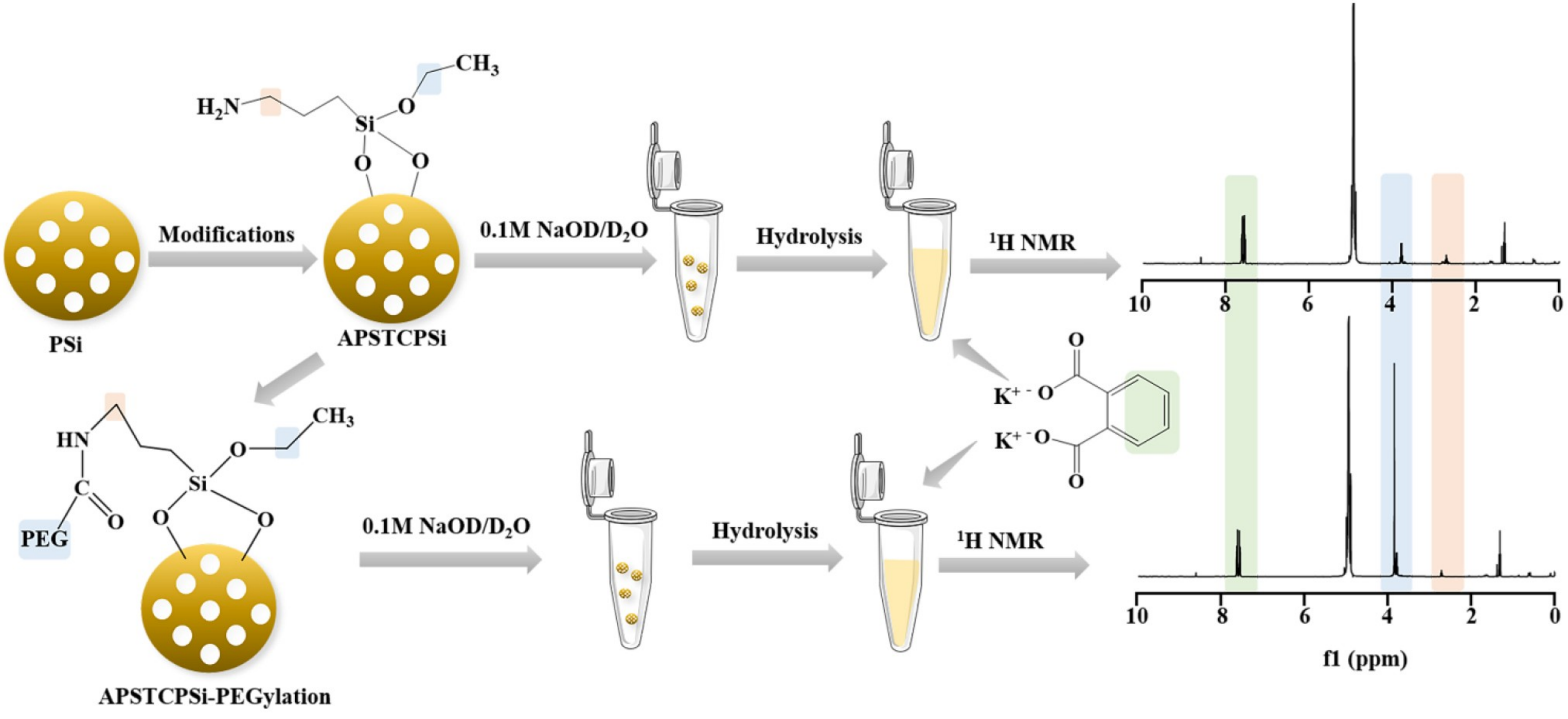
Schematic Diagram of the Functionalization Process of APSTCPSi and APSTCPSi-PEGylation, the Hydrolysis and the Quantitative Analysis of Nanoparticles by Solution ^1^H NMR

**Figure 1:**
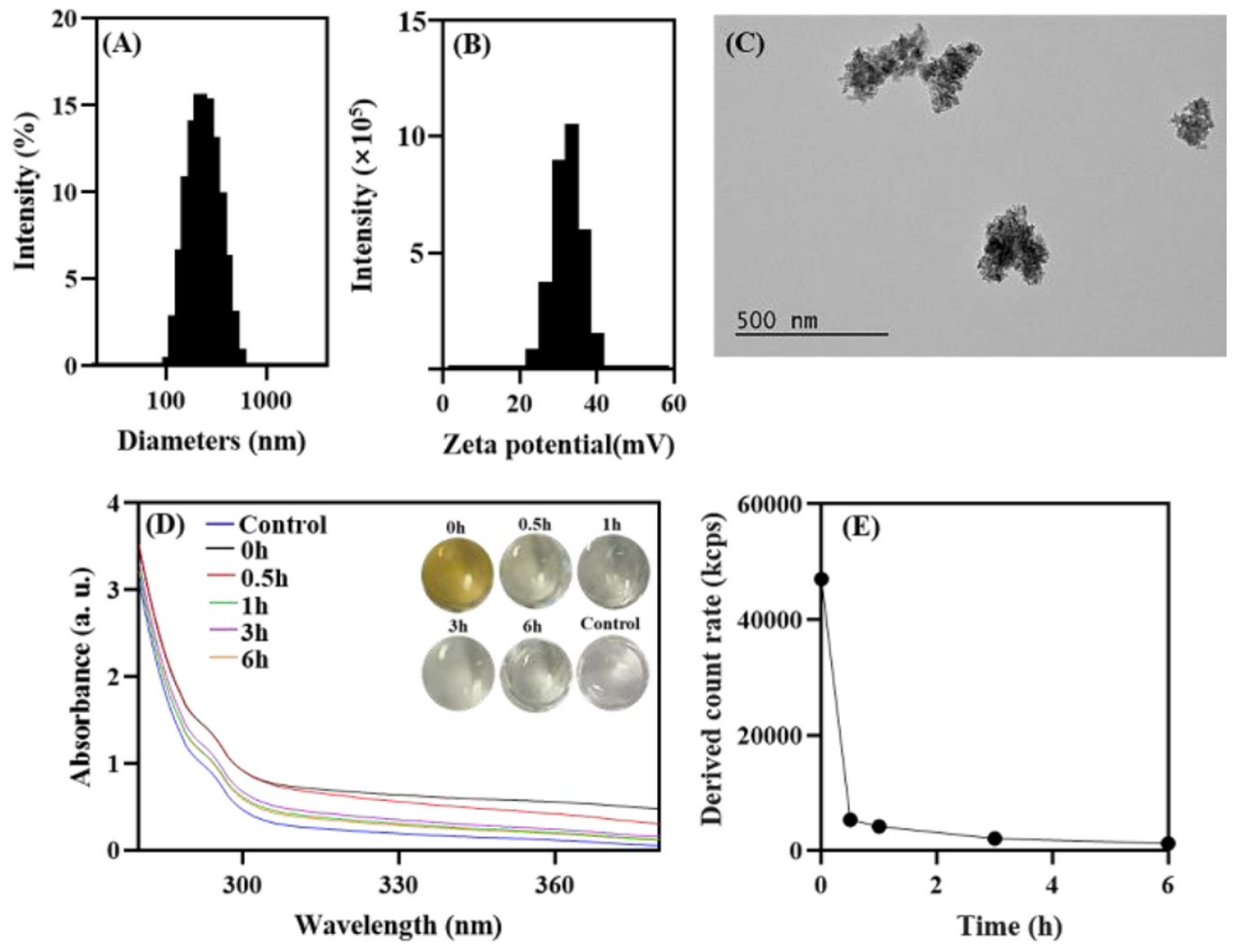
Characteristics and the hydrolysis of APSTCPSi nanoparticles. (A) Hydrodynamic diameter, (B) zeta potential, (C) morphology of APSTCPSi nanoparticles. After incubation in 0.1 M of NaOH for 0.5, 1, 3, and 6 h, (D) UV absorbance, and (E) derived count rate by dynamic light scattering of the APSTCPSi nanoparticle hydrolysis products.

To quantify the amine content, we first optimized the hydrolysis condition for APSTCPSi, to release the surface functional molecules for the detection. Without hydrolysis, the poor hydration of the surface functional groups and the limited mobility made it difficult to analyze by solution NMR directly (Figure S2), similar to the previous report.^24^ Thus, we chose an alkaline solution for the hydrolysis by incubating 0.5 mg of APSTCPSi nanoparticles in 0.1 M of NaOH for different durations (0.5, 1, 3, and 6 h) at 60 °C. The hydrolysis of the nanoparticles was then evaluated by UV absorbance and DLS. As shown in Figure 1D, the particle dispersion lost color dramatically during the first hour of hydrolysis, indicating the fast hydrolytic reaction. After 3 h, the dispersion became almost colorless, suggesting the completion of the hydrolysis. The absorbance of 0.1 M of NaOH was regarded as the control. A similar trend was shown in the UV–vis absorbance spectra. The absorbance gradually decreased with the increase in hydrolysis time. From 3 to 6 h, the sample did not show a clear decrease in the absorbance spectra or displayed any observable change in the color.

We further monitored the count rate of the hydrolysis samples by DLS (Figure 1E). Because the derived count rate is proportional to *cr*^6^ (*c* is the particle concentration and *r* is the particle size), it can be used to detect nanoparticle formation or degradation.^29,30^ After 0.5 h of incubation, the count rate dramatically decreased by almost 1 order of magnitude (from 47041 ± 702 to 5383 ± 43 kcps), revealing the decrease in nanoparticle size or concentration due to degradation. After 1 and 3 h incubation time, the count rates were 4221 ± 34 and 2115 ± 252 kcps, respectively. From 3 to 6 h, the count rate decreased slowly and no obvious nanoparticles trace was observed by the DLS measurements, indicating the hydrolysis of nanoparticles was completed. These results were consistent with the UV-absorbance variation shown in Figure 1D. Thus, this hydrolysis condition (0.1 M of NaOH, 3 h incubation, and 60 °C) was used for the following studies.

Before evaluating the surface amine groups by the solution ^1^H NMR, we identified the ^1^H NMR spectrum of pure APS first, to understand the characteristic peaks and their chemical shifts.^25^ As shown in Figure 2A, we observed the methylene protons on the α-carbon next to the amine groups have characteristic triplet peaks from 2.80 to 2.85 ppm (denoted as peak a in the following discussions). Methylene protons on β- and γ-carbons next to amine groups (peak b and c) showed chemical shifts at 1.63 and 0.62 ppm, respectively. The protons from the ethyl group (peak d and e) were observed at 3.66 and 1.09 ppm, respectively. The chemical shift and the peak splitting correspond well with previous results reported in the literature.^31^

**Figure 2:**
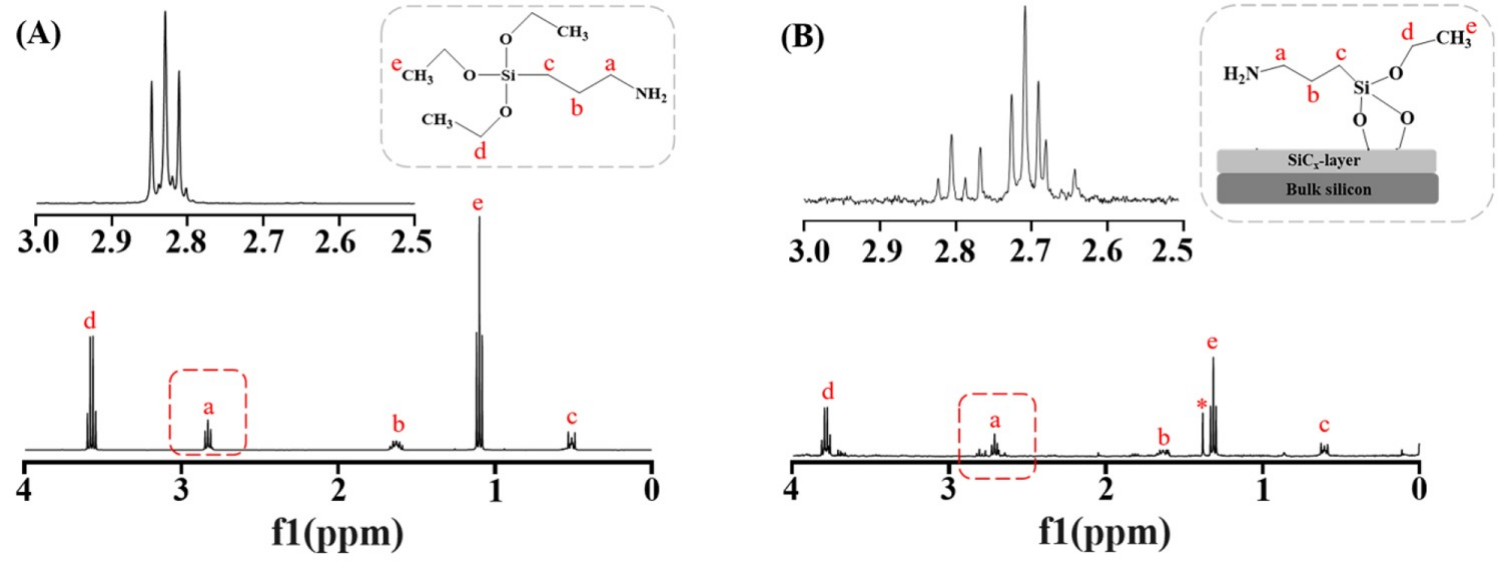
Standard ^1^H NMR spectrum with peaks assigned for the inset molecules. ^1^H NMR spectrum of (A) the (3-aminopropyl) triethoxysilane in D_2_O, and (B) APSTCPSi nanoparticles hydrolysis products in 0.1 M of NaOD/D_2_O. Red circle areas indicate the local magnification of the spectra.

Regarding the APSTCPSi nanoparticles, we hydrolyzed them using the optimized condition and analyzed the spectrum accordingly. As shown in Figure 2B, we found the characteristic peaks of the methylene protons on the α-carbon showed a complex pattern from 2.6 to 2.8 ppm (peak a). The slight decrease in the chemical shift was probably due to alkaline NMR solvent. In 0.1 M of NaOD, the amine group was mostly unprotonated, because the solution pH was higher than its p*K*_a_, whereas in D_2_O, the amine was protonated.^32,33^ Thus, the methylene groups on the α-carbon may have slightly different chemical environments, affecting the chemical shift. The complex pattern (several triplet peaks) may result from incomplete hydrolysis, or different protonation status of the amine groups. We also increased the NaOD concentration to 0.4 M to see if the complex pattern would change with the pH increase. However, we encountered difficulties in NMR probe tuning due to the high ionic concentration. Regarding other characteristic peaks (b–e in Figure 2B) on the degradation products, they are in good agreement with the spectrum of APS (Figure 2A). The incomplete alkoxysilane condensation during the APS modification was confirmed by the presence of the ethoxy groups (peak d and e). Except these identifiable peaks, a sharp singlet peak (marked with * in Figure 2B) at 1.15 ppm indicates some unknown impurity in the particles. These results suggest that it is possible to analyze the APSTCPSi by solution qNMR after dissolution in 0.1 M of NaOD.

After confirming the characteristic peaks of APSTCPSi nanoparticles hydrolysis products, we further improved the quantification results by ^1^H NMR parameter optimization. An essential parameter for the ^1^H NMR is the relaxation delay, during which an excited magnetic state returns to its equilibrium distribution. In our experiments, we mainly focused on the spin–lattice relaxation, also denoted as longitudinal relaxation or *T*_1_, which affects the relative integration between signals.^34^ For quantitative purposes, it is necessary to set the relaxation delay at least 5 times the longest *T*_1_ in the sample between scans to recover 99% of the equilibrium magnetization (Mz).^35^ Therefore, we investigated the relaxation time of the characteristic peaks of APSTCPSi nanoparticles hydrolysis products. In this sample, we also added potassium phthalate (0.5 mg/mL) as the internal standard for quantification purposes. We set a series of relaxation delay (0.01, 0.15, 0.55, 0.125, 2.20, 3.40, 5.00, 6.80, 8.80, 11.30, 13.80, 16.80, and 50.00 s) to determine the *T*_1_ for all the peaks in the spectrum. As shown in Figure S3, the phase of all the peaks gradually changed from negative to positive with the increasing relaxation delay. By fitting the integral data with the equation (*y* = *B* + *F* × exp(−*xG*), we obtained *T*_1_ for all the peaks listed in Table 1. Peaks a–c showed relatively short *T*_1_ (<2 s), whereas peak d and e had slightly longer *T*_1_. The solvent peak exhibited the longest *T*_1_ (16.95 s). To guarantee the sufficient recovery of the equilibrium magnetization between each scan, we applied a 90 s relaxation delay for the following tests to make sure the sufficient recovery of the equilibrium magnetization.

**Table 1:** Curve Fitting Parameters and *T*_1_ for Internal Standard, Characteristic Peaks, And Solvent Peaks for the APSTCPSi Nanoparticle Degradation Product Spectra

peaks	formulation parameters	*T*_1_
internal standard	*B* = 2.27 × 10^5^, *F* = −4.38 × 10^5^, *G* = 0.19	5.08
solvent	*B* = 2.05 × 10^6^*F* = −3.81 × 10^6^, *G* = 0.06	16.95
peak a	*B* = ,2.38 × 10^3^*F* = −4.38 × 10^3^, *G* = 0.61	1.63
peak b	*B* = 2.21 × 10^3^, *F* = −4.10 × 10^3^, *G* = 0.63	1.57
peak c	*B* = 1.43 × 10^5^, *F* = −2.75 × 10^5^, *G* = 0.92	1.08
peak d	*B* = 1.01 × 10^5^, *F* = −1.95 × 10^5^, *G* = 0.15	6.51
peak e	*B* = 3.26 × 10^3^, *F* = −6.03 × 10^3^, *G* = 0.16	6.11

Signal to noise-ratio (S/N) is another essential aspect of NMR experiment as it defines the quality and extent of data that can be obtained, which is extremely important for spectroscopy with low concentration samples, such as surface modification and biological macromolecules.^36^ The S/N can be increased by increasing the number of scans, but at a cost of extending the analysis time. Thus, we explored the relationship between S/N and the number of scans to find a compromise between spectrum quality and experiment length. Specifically, we changed the number of scans from 16 to 256, and we chose the peak a as the object for the S/N analysis.

As shown in Table 2, with 16 scans, the S/N is only 53. Increasing the scan number from 16 to 64 scans led to a significant increase in S/N to 136. However, a further increase to 256 scans only increased S/N to 240. According to the literature, S/N ≥ 20 leads to uncertainty below 5%.^37^ This means even a 16 scan is enough if an estimation with 5% uncertainty is acceptable. S/N ≥ 150 is necessary if the ≤1% uncertainty is required.^37^ In this case, 128 or 256 scans should be used. In our following studies, we chose 256 scans to minimize the uncertainty. After the optimization of both the hydrolysis condition and the ^1^H NMR parameter, we used the optimized protocol to analyze the amine contents on APSTCPSi. We assumed there was a positive correlation between the amine groups on the surface of APSTCPSi nanoparticles and the weight of nanoparticles, as the APSTCPSi nanoparticles were fabricated by covalent conjugation of APS. To verify our hypothesis, we hydrolyzed different amounts of APSTCPSi nanoparticles (0.5, 0.75, 1, 1.25, 1.5, and 2 mg) in 800 μL of 0.1 M of NaOD/D_2_O with internal standard and analyzed the hydrolysis products by the ^1^H NMR. The ^1^H NMR spectra are demonstrated in Figure 3A. All these samples displayed similar peak areas of internal standard (7.51 to 7.64 ppm, 4H from benzene ring of potassium phthalate), as they all contained the same amount of potassium phthalate. This phenomenon also indicates that the interaction between the potassium phthalate and APSTCPSi nanoparticles hydrolysis products was limited, which means the potassium phthalate is a suitable internal standard for this ^1^H NMR analysis and quantitative analysis. Additionally, the increasing peak area was observed in the peak a (2H from methylene protons next to the amine groups), which is attributed to the increasing amount of APSTCPSi nanoparticle hydrolysis products. This result confirms the positive correlation between the amines groups and the amount of APSTCPSi nanoparticles. To further explore this correlation, we normalized the integrated area by the internal standard peaks. We then integrated the area of peak a and applied the curve fitting between the integrated area and the amount of the APSTCPSi nanoparticles. As shown in Figure 3B, the correlation was linear with the *R*^2^ at 0.992 from 0.5 to 2 mg. On the basis of this correlation, we calculated the amount of −NH_2_ groups using eq 1 in the Supporting Information. We found that 1 mg of APSTCPSi nanoparticles contains 0.294 μmol of amine groups. To verify our method, we also used elemental analysis to quantitatively analyze the amine groups on the surface of the APSTCPSi nanoparticles. On the basis of the nitrogen element percentage in the particle (Table S1in the Supporting Information), we calculated that 1 mg of APSTCPSi nanoparticles contains 0.843 μmol of −NH_2_ groups (eq 2 in the Supporting Information). One possible reason for this is the incomplete hydrolysis of APSTCPSi, which underestimated the −NH_2_ amount. The thermal carbonization process in the fabrication of APSTCPSi significantly improved its stability, compared with the PSi with only thermal oxidation treatment.^32^ Thus, the carbonization layer and the stable Si–C bond probably made it difficult to achieve complete degradation in 0.1 M of NaOD even at elevated temperatures.

**Table 2:** S/N of the Peak a with Different Scan Numbers for 1 mg of APSTCPSi Nanoparticles

no. of scans	signal-to-noise ratio (S/N)
16	52.59
64	136.03
128	172.87
256	240.12

**Figure 3:**
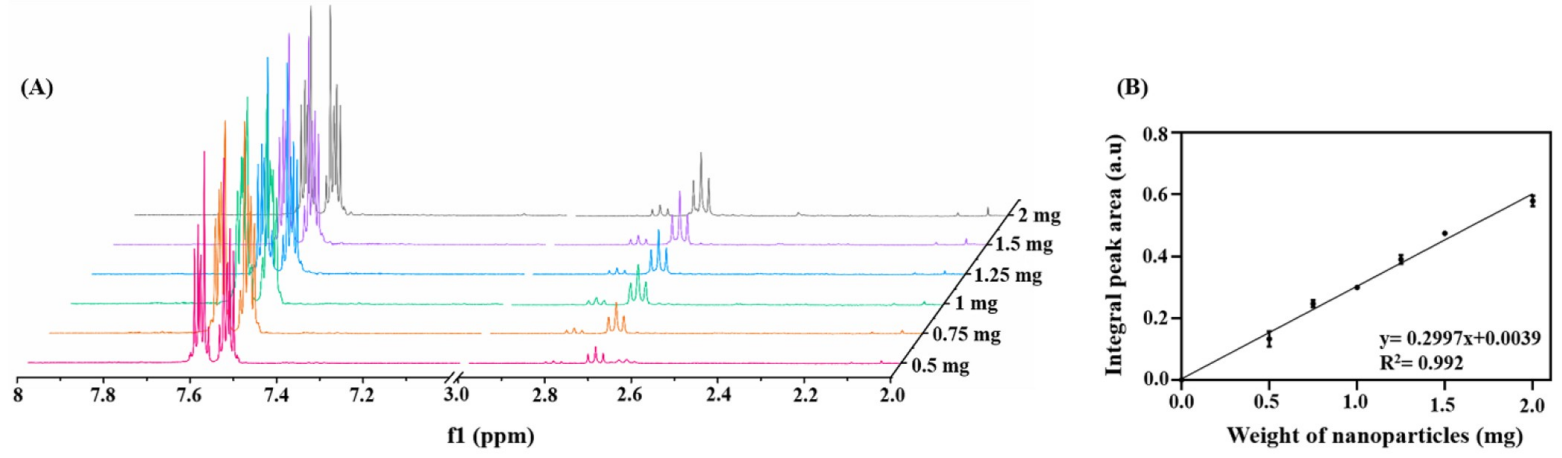
Correlation between amines groups and the amount of APSTCPSi nanoparticles. (A) ^1^H NMR spectra of APSTCPSi nanoparticles with different mass (0.5, 0.75, 1, 1.25, 1.5, and 2 mg), and (B) the curve fitting between the integrated areas of peak a and the mass of the APSTCPSi nanoparticles.

In addition to the amine groups quantification, we further verified our method on APSTCPSi with other functional groups. We chose PEG in this case, because PEGylation is widely used for nanoparticle surface functionalization to increase the stability in biological environments.^38^ Therefore, we first modified the surface of APSTCPSi nanoparticles by poly(ethylene glycol) methyl ether-*N*-hydroxysuccinimide (mPEG-NHS, *M*_w_ = 5000 Da) (Figure 4A). Different molar ratios between the amine groups of APSTCPSi and mPEG-NHS (1:0.1, 1:0.5, 1:1, and 1:2) were used to fabricate the APSTCPSi nanoparticles with different amounts of PEG on the surface. Then these nanoparticles were hydrolyzed and analyzed by our method to explore whether our quantitative method can still successfully analyze the PEGylation on the surface of these nanoparticles.

**Figure 4:**
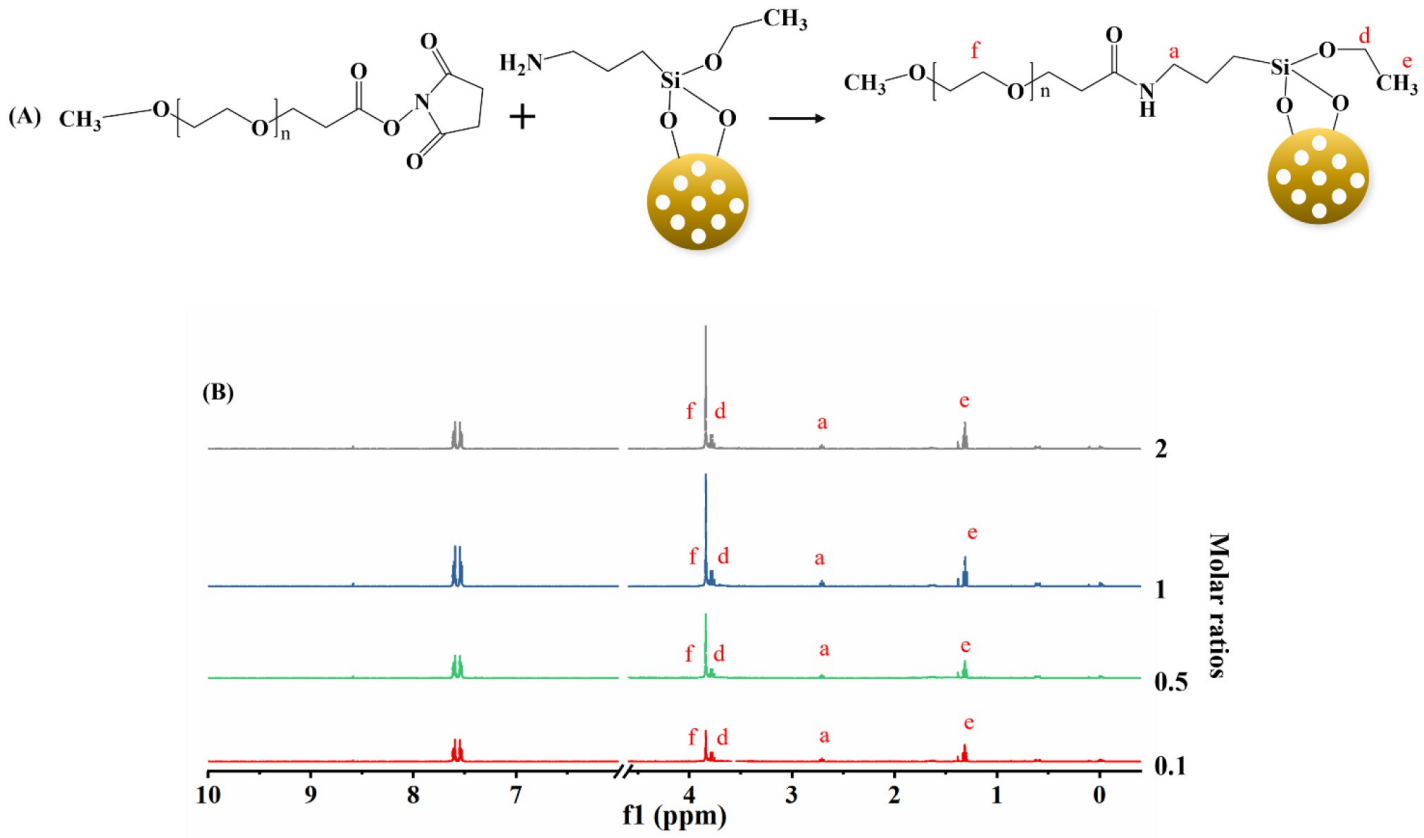
Correlation between amines groups and the mPEG-NHS. (A) Schematic diagram of chemical reactions between mPEG-NHS and APSTCPSi nanoparticles and (B) ^1^H NMR spectra of PEGylated APSTCPSi nanoparticles at different molar ratios (amine groups of APSTCPSi: mPEG-NHS = 1:0.1, 1:0.5, 1:1, and 1:2).

As shown in Figure 4B, the increase in the peak area was observed from 3.70 to 3.90 ppm (−OCH_2_CH_2_O– in the PEG repeating units, denoted as peak f), when increasing the molar ratio of mPEG-NHS in the surface modification reaction. To further quantify this result, we integrated the peak area (3.70 to 3.90 ppm) of the PEG, after normalization by the internal standard. As peak f was partially overlapping with peak d, the integration (3.70 to 3.90 ppm) includes both peaks. To deduce the area of peak d (−CH_2_ from the ethoxy group), we integrated of peak e (−CH_3_ from the same ethoxy group), and calculated by the eqs 3 and 4 in the Supporting Information. As shown in Table 3, when the molar ratios between mPEG-NHS and the amine groups of the nanoparticles increased from 0.1:1 to 2:1, the conjugated PEG increased from 0.010 to 0.025 μmol, whereas the percentage of PEG conjugated decreased from 34.0 to 4.3%. Even at the highest feeding ratio, because of the steric hindrances limiting accessibility to amine groups deep in the smaller pores, only 8.5% amine reacted with PEG. But in terms of the weight percentage, there was 12.5 wt % PEG at the highest feeding ratio, which made a considerable mass fraction in the final particle. These results suggest it is not possible to consume all the surface amine groups even with excessive mPEG-NHS, but the overall PEG coverage could be increased if the PEG feeding ratio is increased. Such PEG coverage data would be valuable for designing PEGylated PSi for biomedical applications.

**Table 3:** Impact of PEG Feeding Ratio on the Final Conjugation, Calculated by qNMR

ratio (amine on APSTCPSi:mPEG-NHS)	*N*_PEG_ (μmol)^a^	*N*_PEG_/*N*_–NH2_ (%)^b^	PEG reaction efficiency (%)^c^	*W*_PEG_/*W*_APSTCPSi_ (%)^d^
1:0.1	0.010	3.4	34.0	5.0
1:0.5	0.015	5.1	10.2	7.5
1:1	0.018	6.1	6.1	9.0
1:2	0.025	8.5	4.3	12.5

a The amount of PEG on the surface of 1 mg of APSTCPSi nanoparticles.

b The percentage of −NH_2_ substituted by PEG.

c The percentage of PEG reacted compared with the feeding PEG in the reaction.

d The weight percentage of PEG on the conjugated APSTCPSi nanoparticles.

In summary, we propose here a new method for APSTCPSi nanoparticles’ surface chemistry quantification by solution ^1^H NMR. The nanoparticles were first degraded in the alkaline solution, making the surface functional groups accessible for NMR detection. After the optimization of the hydrolysis conditions to ensure the degradation, we investigated the key parameters involved in qNMR, including the relaxation delay and the number of scans. The sufficient relaxation delay guaranteed that all signals have relaxed fully between pulses, and the optimized number of scans provided the reliable S/N for the NMR spectra. With an internal standard, we verified the positive correlation between amines groups and the amount of APSTCPSi nanoparticles from 0.5 to 2 mg. Furthermore, we applied this method to characterize the PEGylation of APSTCPSi nanoparticles. When increasing the mPEG-NHS feeding ratio, the conjugated PEG amount increased but the reaction efficiency decreased. With easy sample preparation, high sensitivity, and relatively low cost, the developed method demonstrated here has great potential for PSi-based nanoparticle characterization, complementary to current available quantification methods (TGA and colorimetric assays).

## Supporting Information Available

Experimental details about methods and materials, the NMR results of suspended the APSTCPSi nanoparticles in D_2_O, the relaxation delay optimization, equations for calculation, and element analysis results of APSTCPSi nanoparticles (PDF)

## References

[ref1] Canham L. T. Silicon Quantum Wire Array Fabrication by Electrochemical and Chemical Dissolution of Wafers. Appl. Phys. Lett. 1990, 57 (10), 1046–1048. 10.1063/1.103561.

[ref2] Bimbo L. M.; Sarparanta M.; Santos H. A.; Airaksinen A. J.; Mäkilä E.; Laaksonen T.; Peltonen L.; Lehto V.-P.; Hirvonen J.; Salonen J. Biocompatibility of Thermally Hydrocarbonized Porous Silicon Nanoparticles and Their Biodistribution in Rats. ACS Nano 2010, 4 (6), 3023–3032. 10.1021/nn901657w. 20509673

[ref3] Canham L. T. Bioactive Silicon Structure Fabrication through Nanoetching Techniques. Adv. Mater. 1995, 7 (12), 1033–1037. 10.1002/adma.19950071215.

[ref4] Korhonen E.; Rönkkö S.; Hillebrand S.; Riikonen J.; Xu W.; Järvinen K.; Lehto V.-P.; Kauppinen A. Cytotoxicity Assessment of Porous Silicon Microparticles for Ocular Drug Delivery. Eur. J. Pharm. Biopharm. 2016, 100, 1–8. 10.1016/j.ejpb.2015.11.020. 26686646

[ref5] Layouni R.; Choudhury M. H.; Laibinis P. E.; Weiss S. M. Thermally Carbonized Porous Silicon for Robust Label-Free DNA Optical Sensing. ACS Appl. Bio Mater. 2020, 3 (1), 622–627. 10.1021/acsabm.9b01002. 35019406

[ref6] Kim D.; Kang J.; Wang T.; Ryu H. G.; Zuidema J. M.; Joo J.; Kim M.; Huh Y.; Jung J.; Ahn K. H.; Kim K. H.; Sailor M. J. Two-Photon In Vivo Imaging with Porous Silicon Nanoparticles. Adv. Mater. 2017, 29 (39), 1703309 10.1002/adma.201703309. 28833739

[ref7] Xu R.; Zhang G.; Mai J.; Deng X.; Segura-Ibarra V.; Wu S.; Shen J.; Liu H.; Hu Z.; Chen L.; Huang Y.; Koay E.; Huang Y.; Liu J.; Ensor J. E.; Blanco E.; Liu X.; Ferrari M.; Shen H. An Injectable Nanoparticle Generator Enhances Delivery of Cancer Therapeutics. Nat. Biotechnol. 2016, 34 (4), 414–418. 10.1038/nbt.3506. 26974511 PMC5070674

[ref8] Kang J.; Kim D.; Wang J.; Han Y.; Zuidema J. M.; Hariri A.; Park J.-H.; Jokerst J. V.; Sailor M. J. Enhanced Performance of a Molecular Photoacoustic Imaging Agent by Encapsulation in Mesoporous Silicon Nanoparticles. Adv. Mater. 2018, 30 (27), 1800512 10.1002/adma.201800512. PMC6309700 29782671

[ref9] Kwon E. J.; Skalak M.; Bertucci A.; Braun G.; Ricci F.; Ruoslahti E.; Sailor M. J.; Bhatia S. N. Porous Silicon Nanoparticle Delivery of Tandem Peptide Anti-Infectives for the Treatment of Pseudomonas Aeruginosa Lung Infections. Adv. Mater. 2017, 29 (35), 1701527 10.1002/adma.201701527. PMC5765747 28699173

[ref10] Stead S. O.; McInnes S. J. P.; Kireta S.; Rose P. D.; Jesudason S.; Rojas-Canales D.; Warther D.; Cunin F.; Durand J.-O.; Drogemuller C. J.; Carroll R. P.; Coates P. T.; Voelcker N. H. Manipulating Human Dendritic Cell Phenotype and Function with Targeted Porous Silicon Nanoparticles. Biomaterials 2018, 155, 92–102. 10.1016/j.biomaterials.2017.11.017. 29175084

[ref11] Jin Y.; Kim D.; Roh H.; Kim S.; Hussain S.; Kang J.; Pack C.-G.; Kim J. K.; Myung S.-J.; Ruoslahti E.; Sailor M. J.; Kim S. C.; Joo J. Tracking the Fate of Porous Silicon Nanoparticles Delivering a Peptide Payload by Intrinsic Photoluminescence Lifetime. Adv. Mater. 2018, 30 (35), 1802878 10.1002/adma.201802878. PMC6177232 30003620

[ref12] Mariani S.; Paghi A.; La Mattina A. A.; Debrassi A.; Dähne L.; Barillaro G. Decoration of Porous Silicon with Gold Nanoparticles via Layer-by-Layer Nanoassembly for Interferometric and Hybrid Photonic/Plasmonic (Bio)Sensing. ACS Appl. Mater. Interfaces 2019, 11 (46), 43731–43740. 10.1021/acsami.9b15737. 31644268

[ref13] Almeida P. V.; Shahbazi M.-A.; Mäkilä E.; Kaasalainen M.; Salonen J.; Hirvonen J.; Santos H. A. Amine-Modified Hyaluronic Acid-Functionalized Porous Silicon Nanoparticles for Targeting Breast Cancer Tumors. Nanoscale 2014, 6 (17), 10377–10387. 10.1039/C4NR02187H. 25074521 PMC4234906

[ref14] Li W.; Liu Z.; Fontana F.; Ding Y.; Liu D.; Hirvonen J. T.; Santos H. A. Tailoring Porous Silicon for Biomedical Applications: From Drug Delivery to Cancer Immunotherapy. Adv. Mater. 2018, 30 (24), 1703740 10.1002/adma.201703740. 29534311

[ref15] Näkki S.; Rytkönen J.; Nissinen T.; Florea C.; Riikonen J.; Ek P.; Zhang H.; Santos H. A.; Närvänen A.; Xu W.; Lehto V.-P. Improved Stability and Biocompatibility of Nanostructured Silicon Drug Carrier for Intravenous Administration. Acta Biomater. 2015, 13, 207–215. 10.1016/j.actbio.2014.11.019. 25463492

[ref16] Ferreira M. P. A.; Ranjan S.; Kinnunen S.; Correia A.; Talman V.; Mäkilä E.; Barrios-Lopez B.; Kemell M.; Balasubramanian V.; Salonen J.; Hirvonen J.; Ruskoaho H.; Airaksinen A. J.; Santos H. A. Drug-Loaded Multifunctional Nanoparticles Targeted to the Endocardial Layer of the Injured Heart Modulate Hypertrophic Signaling. Small 2017, 13 (33), 1701276 10.1002/smll.201701276. 28714245

[ref17] Kunc F.; Balhara V.; Sun Y.; Daroszewska M.; Jakubek Z. J.; Hill M.; Brinkmann A.; Johnston L. J. Quantification of Surface Functional Groups on Silica Nanoparticles: Comparison of Thermogravimetric Analysis and Quantitative NMR. Analyst 2019, 144 (18), 5589–5599. 10.1039/C9AN01080G. 31418443

[ref18] Sun N.; Meng X.; Xiao Z. Functionalized Si3N4 Nanoparticles Modified with Hydrophobic Polymer Chains by Surface-Initiated Atom Transfer Radical Polymerization (ATRP). Ceram. Int. 2015, 41 (10), 13830–13835. 10.1016/j.ceramint.2015.08.068.

[ref19] Burleigh M. C.; Markowitz M. A.; Spector M. S.; Gaber B. P. Amine-Functionalized Periodic Mesoporous Organosilicas. Chem. Mater. 2001, 13 (12), 4760–4766. 10.1021/cm0105763.

[ref20] Burleigh M. C.; Markowitz M. A.; Jayasundera S.; Spector M. S.; Thomas C. W.; Gaber B. P. Mechanical and Hydrothermal Stabilities of Aged Periodic Mesoporous Organosilicas. J. Phys. Chem. B 2003, 107 (46), 12628–12634. 10.1021/jp035189q.

[ref21] Park J.-H.; Gu L.; von Maltzahn G.; Ruoslahti E.; Bhatia S. N.; Sailor M. J. Biodegradable Luminescent Porous Silicon Nanoparticles for in Vivo Applications. Nat. Mater. 2009, 8 (4), 331–336. 10.1038/nmat2398. 19234444 PMC3058936

[ref22] Singh S.; Roy R. The Application of Absolute Quantitative 1H NMR Spectroscopy in Drug Discovery and Development. Expert Opin. Drug Discovery 2016, 11 (7), 695–706. 10.1080/17460441.2016.1189899. 27187052

[ref23] Baccile N.; Laurent G.; Bonhomme C.; Innocenzi P.; Babonneau F. Solid-State NMR Characterization of the Surfactant–Silica Interface in Templated Silicas: Acidic versus Basic Conditions. Chem. Mater. 2007, 19 (6), 1343–1354. 10.1021/cm062545j.

[ref24] Crucho C. I. C.; Baleizão C.; Farinha J. P. S. Functional Group Coverage and Conversion Quantification in Nanostructured Silica by 1H NMR. Anal. Chem. 2017, 89 (1), 681–687. 10.1021/acs.analchem.6b03117. 28105822

[ref25] Hristov D. R.; Rocks L.; Kelly P. M.; Thomas S. S.; Pitek A. S.; Verderio P.; Mahon E.; Dawson K. A. Tuning of Nanoparticle Biological Functionality through Controlled Surface Chemistry and Characterisation at the Bioconjugated Nanoparticle Surface. Sci. Rep. 2015, 5 (1), 17040 10.1038/srep17040. 26621190 PMC4664868

[ref26] Sun Y.; Kunc F.; Balhara V.; Coleman B.; Kodra O.; Raza M.; Chen M.; Brinkmann A.; Lopinski G. P.; Johnston L. J. Quantification of Amine Functional Groups on Silica Nanoparticles: A Multi-Method Approach. Nanoscale Adv. 2019, 1 (4), 1598–1607. 10.1039/C9NA00016J. 36132607 PMC9417554

[ref27] Qi S.; Zhang P.; Ma M.; Yao M.; Wu J.; Mäkilä E.; Salonen J.; Ruskoaho H.; Xu Y.; Santos H. A.; Zhang H. Cellular Internalization–Induced Aggregation of Porous Silicon Nanoparticles for Ultrasound Imaging and Protein-Mediated Protection of Stem Cells. Small 2019, 15 (1), 1804332 10.1002/smll.201804332. 30488562

[ref28] Martinez J. O.; Boada C.; Yazdi I. K.; Evangelopoulos M.; Brown B. S.; Liu X.; Ferrari M.; Tasciotti E. Short and Long Term, In Vitro and In Vivo Correlations of Cellular and Tissue Responses to Mesoporous Silicon Nanovectors. Small 2013, 9 (9–10), 1722–1733. 10.1002/smll.201201939. 23255523 PMC3707147

[ref29] Wang S.; Wannasarit S.; Figueiredo P.; Li J.; Correia A.; Xia B.; Wiwattanapatapee R.; Hirvonen J.; Liu D.; Li W.; Santos H. A. Superfast and Controllable Microfluidic Inking of Anti-Inflammatory Melanin-like Nanoparticles Inspired by Cephalopods. Mater. Horiz. 2020, 7 (6), 1573–1580. 10.1039/D0MH00014K.

[ref30] Shang J.; Gao X. Nanoparticle Counting: Towards Accurate Determination of the Molar Concentration. Chem. Soc. Rev. 2014, 43 (21), 7267–7278. 10.1039/C4CS00128A. 25099190 PMC4188810

[ref31] Miyata T.; Kawamura A.; Meotoiwa T.; Matsumoto M.; Uragami T. Synthesis of Novel Nucleobase-Terminated Organosilane and Its Self-Assembly on a Substrate. Polym. J. 2012, 44 (6), 625–631. 10.1038/pj.2012.41.

[ref32] Mäkilä E.; Bimbo L. M.; Kaasalainen M.; Herranz B.; Airaksinen A. J.; Heinonen M.; Kukk E.; Hirvonen J.; Santos H. A.; Salonen J. Amine Modification of Thermally Carbonized Porous Silicon with Silane Coupling Chemistry. Langmuir 2012, 28 (39), 14045–14054. 10.1021/la303091k. 22967052

[ref33] Hiramatsu H.; Osterloh F. E. PH-Controlled Assembly and Disassembly of Electrostatically Linked CdSe–SiO2 and Au–SiO2 Nanoparticle Clusters. Langmuir 2003, 19 (17), 7003–7011. 10.1021/la034217t.

[ref34] Hitomi Y.; Aoki K.; Miyachi R.; Ohyama J.; Kodera M.; Tanaka T.; Sugihara F. Gold Nanoparticles Coated with Manganese–Porphyrin That Effectively Shorten the Longitudinal Relaxation Time of Water Molecules Depending on the Particle Size. Chem. Lett. 2014, 43 (12), 1901–1903. 10.1246/cl.140812.

[ref35] Gowland P. A.; Stevenson V. L. *T*_1_: The Longitudinal Relaxation Time. In Quantitative MRI of the Brain; Wiley, 2003; pp 111–141. 10.1002/0470869526.ch5

[ref36] Hyberts S. G.; Robson S. A.; Wagner G. Exploring Signal-to-Noise Ratio and Sensitivity in Non-Uniformly Sampled Multi-Dimensional NMR Spectra. J. Biomol. NMR 2013, 55 (2), 167–178. 10.1007/s10858-012-9698-2. 23274692 PMC3570699

[ref37] Malz F.; Jancke H. Validation of Quantitative NMR. J. Pharm. Biomed. Anal. 2005, 38 (5), 813–823. 10.1016/j.jpba.2005.01.043. 15893442

[ref38] Suk J. S.; Xu Q.; Kim N.; Hanes J.; Ensign L. M. PEGylation as a Strategy for Improving Nanoparticle-Based Drug and Gene Delivery. Adv. Drug Delivery Rev. 2016, 99, 28–51. 10.1016/j.addr.2015.09.012. PMC4798869 26456916

