# Supplementary material for: Quantitative Analysis of Porous Silicon Nanoparticles Functionalization by 1H NMR
Source: ACS Biomater Sci Eng. 2021 Jul 22;8(10):4132–9. doi: 10.1021/acsbiomaterials.1c00440 (PMC9554871; doi:10.1021/acsbiomaterials.1c00440)
Supplement: Supplementary file 1 — ab1c00440_si_001.pdf [file ab1c00440_si_001.pdf]

Supporting Information

Quantitative Analysis of Porous Silicon Nanoparticles Functionalization by ^{1}H NMR

Ruoyu Cheng[†, 1], Shiqi Wang[†, *, 1], Karina Moslova[¶], Ermei Mäkilä[‖], Jarno Salonen[‖], Jiachen Li[†, §], Jouni Hirvonen[†], Bing Xia[§], Hélder A. Santos[†, ‡, *]

[+] Drug Research Program, Division of Pharmaceutical Chemistry and Technology, Faculty of Pharmacy, University of Helsinki, FI-00014 Helsinki, Finland

[‡] Helsinki Insititute of Life Science, HiLIFE, University of Helsinki, FI-00014 Helsinki, Finland

[¶]Department of Chemistry, Faculty of Science, University of Helsinki, FI-00014 Helsinki, Finland

[§]College of Science Key Laboratory of Forest Genetics & Biotechnology (Ministry of Education of China), Nanjing Forestry University, Nanjing 210037, P. R. China

[//] Laboratory of Industrial Physics, Department of Physics and Astronomy, University of Turku, FI-20014 Turku, Finland

[1]These authors contributed equally to this work.

EXPERIMENTAL SECTION

Materials

D_2O (99.9 atom % D, CAS: 7789-20-0), 3 - (aminopropyl) triethoxysilane (99%, CAS: 919-30-2), NaOD (40 wt.-% in D_2O, 99 atoms % D, CAS: 14014-06-3) and potassium phthalate (CAS: 4409-98-7) were purchased from Sigma-Aldrich® (USA). methyl ether-N-hydroxysuccinimide (mPEG-NHS, M_W 5000 Da) was purchased from NANOCS, Inc.(USA). Silicon wafers were purchased from Siegert Wafer GmbH (Germany).

APSTCPSi Nanoparticles Fabrication

The nanoparticles were produced by following the fabrication steps outlined previously.[1-3] In detail, multilayer porous silicon (PSi) films were electrochemically anodized on monocrystalline, boron doped p^+-type Si (100) wafers of 0.01–0.02 Ωcm resistivity. Electrolyte used in the process was a 1:1 (vol.) solution of hydrofluoric acid (HF, 38%) and absolute ethanol (EtOH). The anodization current profile repeated a cycle of low and high current density periods to create fracture planes on the PSi layer to assist in the milling process. The PSi multilayer was finally lifted off from the wafer as a freestanding film utilizing an electropolishing current pulse. The PSi films were initially stabilized by thermal carbonization using acetylene (C_2H_2) as follows. The PSi films were dried under N_2 flow in a quartz tube for at least 30 min to remove residual moisture and oxygen at room temperature. Following the drying, acetylene was added to the gas flow at a ratio of 1:1 (vol.) N_2/C_2H_2 for 15 min at room temperature. Under

the N_2/C_2H_2 flow, the tube was placed in a furnace for 15 min at 500°C, after which the films were allowed to cool back to room temperature under N_2 flow. Finally, N_2/C_2H_2 flow was added at room temperature for 10 min before thermally annealing the films at 820°C for 10 min under N_2 flow. The obtained thermally carbonized PSi (TCPSi) films were then cooled back to room temperature under N_2 flow.

The non-stoichiometric SiC surface structure enabled functionalization of the TCPSi films through generation of –OH terminal groups on the surface by immersing the films briefly into HF solution (1:1 (vol.) HF/EtOH).[4] Following this, the films were silanized for 1 h using a 10 vol-% 3-(aminopropyl)triethoxysilane (APTES) – anhydrous toluene solution at room temperature. After removal of the excess silane, the films were dried for 16 h at 105°C. The aminopropylsilane-terminated TCPSi films (APSTCPSi) were then milled in a ball mill into nanoparticles using a 5 vol-% APTES-toluene solution as the milling medium. Size selection of the APSTCPSi nanoparticles was done with centrifugation using EtOH as the particle dispersant.

APSTCPSi Nanoparticles Characterization

The morphology of APSTCPSi was investigated by a transmission electron microscope (TEM, Tecnai F12, FEI Company, USA). The hydrodynamic diameter (Z-average) and zeta-potential of the nanoparticles were analyzed by a Zetasizer Nano ZS (Malvern Instruments, UK) at 25 °C. The porosity of the particle was analyzed with nitrogen sorption at −196 °C using Tristar 3000 (Micromeritics Inc., USA). Specific surface area was calculated from the isotherm (**Figure S1**) using Brunauer-Emmett-

Teller method and the pore volume was estimated as the total adsorbed amount at a relative pressure of 0.97. Average pore diameter was calculated by assuming the pore shape as cylindrical.

APSTCPSi Nanoparticles Hydrolysis

To optimize the hydrolysis condition of APSTCPSi nanoparticles, 0.5 mg of nanoparticles were dried in the oven at 40 ℃ overnight. Then, the nanoparticles were incubated in the 0.1 M of NaOH solution at 60 ℃ water bath for different time-points (0.5, 1, 3, and 6 h). At each time-point, samples were collected and measured by a Zetasizer Nano ZS. Moreover, these samples were also investigated by ultraviolet–visible absorbance at 250−800 nm by a Varioskan Flash fluorometer (Thermo Fisher Scientific, USA). In addition, a digital camera was used to follow the general hydrolysis of the samples.

NMR Spectroscopy

Solution ^{1}H proton NMR data was collected by a Bruker Avance III 400 spectrometer (Bruker BioSpin GmbH, Rheinstetten, Germany), operating at 400 MHz. The following samples, 1 mg of nanoparticles dispersed into 800 μL of D_2O, 1mg of nanoparticles dissolved in 800 μL of 0.1 M of NaOD/D_2O, methoxypolyethylene glycol acetic acid N-succinimidyl ester (mPEG-NHS) dissolved in 800 μL of D_2O, (3-aminopropyl) triethoxysilane dissolved in 800 μL of D_2O, were all performed using standard ^{1}H NMR protocol (64 scans, 2 dummy scans, 30° pulse, 1 s relaxation delay,

10 μs pulse width, 5.45 s acquisition time, 15 ppm sweep width, 6.5 ppm O^1).

To identify the parameters for qNMR, the spin-lattice relaxation (T_1) was optimized. Specifically, a known amount of potassium phthalate (0.5 mg/mL) was added in 0.1 M of NaOD/D_2O as an internal standard. Then 2 mg of nanoparticles were separately dissolved in 800 μL of 0.1 M of NaOD/D_2O, as described above. The T_1 was measured by running single experiments, changing the delay values (τ = 0.01, 0.15, 0.55, 0.125, 2.20, 3.40, 5.00, 6.80, 8.80, 11.30, 13.80, 16.80, and 50.00 s).

The number of scans was also optimized. Specifically, 1 mg of APSTCPSi nanoparticles were dissolved in 800 μL of 0.1 M of NaOD/D_2O with internal standard. Then different number of scans (from 16 to 256) were performed. Signal noise/ratio (S/N) was used to determine the optimized number of scans.

Different amount (0.5, 0.75, 1, 1.25, 1.5, and 2 mg) of APSTCPSi nanoparticles (in triplicate) were incubated in 800 μL of 0.1 M of NaOD/D_2O with internal standard at 60 ℃ for 3 h. Until the nanoparticles were totally dissolved, they were measured in the NMR experiments using a 90° pulse scan program with optimized qNMR parameters (256 scans, 90 s relaxation delay).

APSTCPSi Nanoparticles PEGylation and Quantitative NMR Spectroscopy

APSTCPSi nanoparticles and mPEG-NHS were dissolved into 1 mL of HBSS (pH 7.4) with different molar ratios (1:0.1, 1:0.5, 1:1, and 1:2, amine:NHS) with constant stirring. Then the reaction was performed for 3 h at room temperature. After that, the nanoparticles were centrifuged for 5 min (15000*g*) and washed with deionized (DI)

water for three times. After drying overnight at 40 ℃ in the oven, these nanoparticles were dissolved in 800 μL of 0.1 M of NaOD/D_2O with internal standard at 60 ℃ for 3 h and further analyzed, using the same qNMR protocol described above.

NMR Data Processing and Calculation of the –NH_2 functional groups

All the NMR spectra were processed by MestReNova software. After Fourier transformation, the chemical shift was referenced by the residual water signal (a broad peak centered at 4.79 ppm). The phase was corrected automatically first, followed manual adjustment for specific peaks if the automatic correction was not satisfying. The baseline was corrected by fifth-order polynomial fit with manually adjusted filter.

To calculate the amine groups on APSTCPSi nanoparticles, we used the integral data from Figure 3a, and calculated by the Eq. (1) below:

$$\frac{\frac{\text{Area}_{\text{Internal standard}}}{4}}{\text{N}_{\text{Internal standard}}} = \frac{\frac{\text{Area}_{\text{peak a}}}{2}}{\text{N}_{-\text{NH}_2}} \qquad (1)$$

where, Area $_{\text{Internal standard}}$ and Area $_{\text{peak a}}$ are the integral area of the internal standard peak (4H from benzene ring of potassium phthalate) and peak a (2H from methylene protons next to the amine groups), $N_{\text{Internal standard}}$ is the molar amount of the internal standard, and $N_{\text{-NH2}}$ is the molar amount of -NH_2.

Elemental analysis of APSTCPSi Nanoparticles

Automatic elemental analyzer vario MICRO cube (HANAU Elementar Analysensysteme GmbH, Germany) was used for the determination of carbon as CO_2, hydrogen as H_2O, nitrogen as N_2 and sulfur as SO_2 in the sample. N_2 is not adsorbed in

the adsorption column and is the first measuring component to enter the thermal conductivity detector (TCD). The adsorption column is heated stepwise to desorption temperatures of the CO_2 (60 °C), H_2O (140 °C) and SO_2 (210 °C). The samples were analyzed in triplicates and the results are shown in **Table S1** below.

Table S1. The element analysis results of APSTCPSi nanoparticles.

Weight (mg)	N %	C %	H %
1.76 ± 0.02	1.18 ± 0.11	18.36 ± 0.63	2.18 ± 0.04

The average amount of APSTCPSi nanoparticles was 1.76 ± 0.02 mg, and the amount of N-% was 1.18 ± 0.11. Thus, the amount of N was 0.02 mg, thus the moles number of N was 0.148 μmol for 1.76 mg of APSTCPSi nanoparticles, calculated using Eq. (2):

$$N_{-NH_2}(mmol) = \frac{Weight_{APSTCPSi} \times N\%}{14} \tag{2}$$

NMR Data Processing and Calculation of the PEGylation functional groups

To calculate the PEG conjugated on the APSTCPSi nanoparticles, we used the integral data from **Figure 4b**, and calculated using Eq. (3):

$$\frac{\frac{\text{Area}_{\text{Internal standard}}}{4}}{\text{N}_{\text{Internal standard}}} = \frac{\frac{\text{Area}_{\text{peak f}}}{4 \times \frac{5000}{44}}}{\text{N}_{\text{PEG}}} \tag{3}$$

where, Area $_{\text{Internal standard}}$ is the integral area of internal standard peak (4H from benzene ring of potassium phthalate), Area $_{\text{peak f}}$ is the integral area of PEG repeating unit (peak f, $-OCH_2CH_2O-$), 5000 is the molecular weight of the mPEG-NHS, 44 means the molecular weight of the repeating units, N $_{\text{Internal standard}}$ is the molar amount of the internal standard, and N $_{\text{PEG}}$ is the molar amount of PEG.

However, because in the spectra shown in **Figure 4b**, peak f is overlapping with peak d (–OCH_2 from the ethoxy group) and the integration was affected accordingly. Then we made the integration of peak e (–OCH_2CH_3 from the same ethoxy group), and deduce the area of peak d from Area $_{\text{peak f+d}}$ by using Eq. (4):

$$\text{Area}_{\text{peak f}} = \text{Area}_{\text{peak f + d}} - \frac{2}{3} \times \text{Area}_{\text{peak e}} \quad (4)$$

Taken altogether the results from Equation 4 and back to Equation 3, the molar amount of PEG was calculated accordingly.

REFERENCE

1. Bimbo, L. M.; Sarparanta, M.; Santos, H. A.; Airaksinen, A. J.; Mäkilä, E.; Laaksonen, T.; Peltonen, L.; Lehto, V.-P.; Hirvonen, J.; Salonen, J. Biocompatibility of Thermally Hydrocarbonized Porous Silicon Nanoparticles and Their Biodistribution in Rats. *ACS Nano* **2010**, *4* (6), 3023–3032. https://doi.org/10.1021/nn901657w.

2. Mäkilä, E.; Bimbo, L. M.; Kaasalainen, M.; Herranz, B.; Airaksinen, A. J.; Heinonen, M.; Kukk, E.; Hirvonen, J.; Santos, H. A.; Salonen, J. Amine Modification of Thermally Carbonized Porous Silicon with Silane Coupling Chemistry. *Langmuir* **2012**, *28* (39), 14045–14054. https://doi.org/10.1021/la303091k.

3. Shahbazi, M.-A.; Almeida, P. V; Mäkilä, E.; Correia, A.; Ferreira, M. P. A.; Kaasalainen, M.; Salonen, J.; Hirvonen, J.; Santos, H. A. Poly(Methyl Vinyl Ether- Alt -Maleic Acid)-Functionalized Porous Silicon Nanoparticles for Enhanced Stability and Cellular Internalization. *Macromol. Rapid Commun.* **2014**, *35* (6), 624–629. https://doi.org/10.1002/marc.201300868.

4. Dhar, S.; Seitz, O.; Halls, M. D.; Choi, S.; Chabal, Y. J.; Feldman, L. C. Chemical Properties of Oxidized Silicon Carbide Surfaces upon Etching in Hydrofluoric Acid. *J. Am. Chem. Soc.* **2009**, *131* (46), 16808–16813. https://doi.org/10.1021/ja9053465.

SUPPLEMENTARY FIGURES

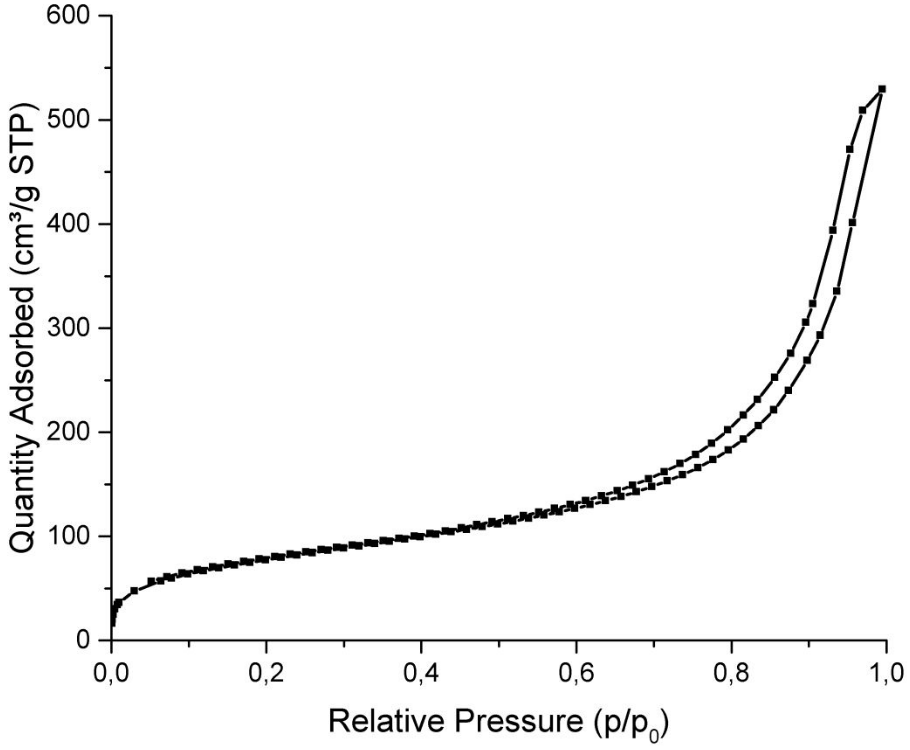

Figure S1. Nitrogen sorption isotherm of APSTCPSi nanoparticles.

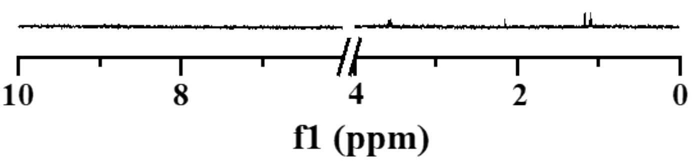

Figure S2. The NMR results of suspended APSTCPSi nanoparticles in D_2O.

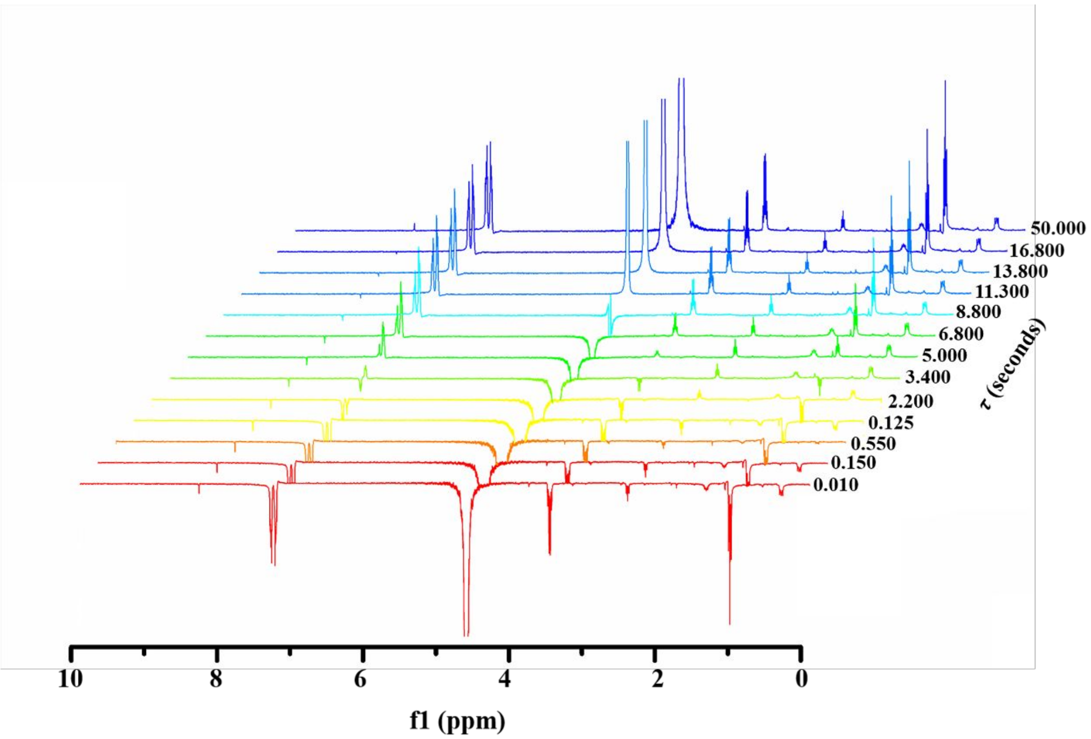

Figure S3. The original NMR spectra of the relaxation delay optimization experiment.
